# The evolving role of social media in enhancing quality of life: a global perspective across 10 countries

**DOI:** 10.1186/s13690-023-01222-z

**Published:** 2024-03-06

**Authors:** Roy Rillera Marzo, Hana W. Jun Chen, Absar Ahmad, Hui Zhu Thew, Ja Shen Choy, Chee Han Ng, Chen Loong Alyx Chew, Petra Heidler, Isabel King, Rajeev Shrestha, Farzana Rahman, Jehan Akhter Rana, Tornike Khoshtaria, Arian Matin, Nugzar Todua, Burcu Küçük Biçer, Erwin Faller, Randy A. Tudy, Aries Baldonado, Criselle Angeline Penamante, Rafidah Bahari, Delan Ameen Younus, Zjwan Mohammed Ismail, Masoud Lotfizadeh, Shehu Muhammad Hassan, Rahamatu Shamsiyyah Iliya, Asari E. Inyang, Theingi Maung Maung, Win Myint Oo, Ohnmar Myint, Anil Khadka, Swosti Acharya, Soe Soe Aye, Thein Win Naing, Myat Thida Win, Ye Wint Kyaw, Pramila Pudasaini Thapa, Josana Khanal, Sudip Bhattacharya, Khadijah Abid, Mochammad Fahlevi, Mohammed Aljuaid, Radwa Abdullah El-Abasir, Mohamed E. G. Elsayed

**Affiliations:** 1 Faculty of Humanities and Health Sciences, Curtin University, Miri, Malaysia; 2 Global Public Health, Jeffrey Cheah School of Medicine and Health Sciences, Monash University Malaysia, Subang Jaya, Selangor Malaysia; 3 International Medical School, Management and Science University, Shah Alam, Selangor 40610 Malaysia; 4 College of Veterinary Science and Animal Husbandry, Birsa Agricultural University, Ranchi, Jharkhand 834006 India; 5 Department of Family Medicine, Faculty of Medicine and Health Sciences, Universiti Putra Malaysia, Serdang, Selangor Malaysia; 6 Faculty of Medicine and Health Sciences, Universiti Putra Malaysia, Serdang, Selangor Malaysia; 7 Institute International Trade and Sustainable Economy, University of Applied Sciences Krems, Krems an der Donau, Austria; 8 Department of Health Sciences, St. Pölten University of Applied Sciences, St. Pölten, Austria; 9 Department of Exercise Physiology, School of Health, University of the Sunshine Coast, Sippy Downs, QLD Australia; 10 Palliative Care and Chronic Disease, Green Pastures Hospital, PO Box 28, Pokhara, Province Gandaki 33700 Nepal; 11 Department of Research & Administration, Bangladesh National Nutrition Council, Mohakhali, Dhaka, Bangladesh; 12 Department of Coordination, National Nutrition Council, Mohakhali, Dhaka, Bangladesh; 13 Faculty of Healthcare Economics and Management, University Geomedi, Tbilisi, Georgia; 14 School of Business, International Black Sea University, Tbilisi, Georgia; 15 School of Economics and Business, Tbilisi State University, Tbilisi, Georgia; 16 Department of Medical Education and Informatics, Gazi University Faculty of Medicine, Ankara, Turkey; 17 Pharmacy Department, School of Allied Health Sciences, San Pedro College, Davao City, Philippines; 18 Faculty of Health and Social Sciences, Bournemouth University, Poole, UK; 19 Faculty of the College of Education, University of Southeastern Philippines, Davao City, Philippines; 20 College of Nursing, Saint Alexius College, Koronadal City, Philippines; 21 Department of Psychology, College of Science, University of Santo Tomas, Manila, Philippines; 22 Department of Clinical Epidemiology, Faculty of Medicine and Surgery, University of Santo Tomas, Manila, Philippines; 23 Department of Psychiatry, Faculty of Medicine, University of Cyberjaya, Cyberjaya, Malaysia; 24 Department of Medical Microbiology, College of Medicine, University of Duhok, Duhok, Iraq; 25 Department of Medical Laboratory Technology, Technical Health and Medical College, Erbil Polytechnique University, Erbil, Iraq; 26 Social Determinants of Health Research Center, Shahrekord University of Medical Sciences, Shahrekord, Iran; 27 Department of Biochemistry, Faculty of Life Sciences, Ahmadu Bello University, Zaria, Nigeria; 28 Department of Public Health, Distance Learning Centre, Ahmadu Bello University, Zaria, Nigeria; 29 School of Public Health, Imperial College London, London, UK; 30 Asian Institute of Medicine, Science and Technology, Bedong, Kedah Malaysia; 31 ASEAN Institute for Health Development, Mahidol University, Salaya, Thailand; 32 Regional Public Health Department, Ayeyarwady Region, Pathein, Myanmar; 33 Department of Public Health Modern Technical College Affiliated to Pokhara University, Lalitpur, Nepal; 34 Manmohan Cardiothoracic Vascular and Transplant Centre, Tribhuvan University Teaching Hospital, Kathmandu, 44600 Nepal; 35 Department of Paediatrics, RCSI Program Perdana University, Kuala Lumpur, Malaysia; 36 Department of Community Medicine, Faculty of Medicine, University of Cyberjaya, Cyberjaya, Malaysia; 37 Department of Internal Medicine, University of Cyberjaya, Cyberjaya, Malaysia; 38 Faculty of Medicine, Nursing and Health Sciences, SEGi University, Petaling Jaya, Malaysia; 39 Life Skill Education Institutes/Yeti Health Science Academy, Kathmandu, Nepal; 40 Department of Public Health (Purbanchal University), Kathmandu, Nepal; 41 Department of Community and Family Medicine, All India Institute of Medical Sciences, Deoghar, Jharkhand India; 42 Department of Ophthalmology and Visual Sciences, Aga Khan University Hospital, Karachi, Pakistan; 43 Management Department, BINUS Online Learning, Bina Nusantara University, Jakarta, 11480 Indonesia; 44 Department of Health Administration, College of Business Administration, King Saud University, Riyadh, Saudi Arabia; 45 Nuffield Department of Population Health, University of Oxford Richard Doll Building, Old Road Campus, Oxford, OX3 7LF UK; 46 Department of Psychiatry and Psychotherapy III, University of Ulm, Ulm, Germany; 47 Department of Psychiatry, School of Medicine and Health Sciences, Carl von Ossietzky University Oldenburg, Oldenburg, Germany

**Keywords:** Social media needs, Quality of life, Affective needs, Georgia, Austria, Epidemiology, Determinants

## Abstract

**Background:**

Excessive or inappropriate use of social media has been linked to disruptions in regular work, well-being, mental health, and overall reduction of quality of life. However, a limited number of studies documenting the impact of social media on health-related quality of life (HRQoL) are available globally.

**Aim:**

This study aimed to explore the perceived social media needs and their impact on the quality of life among the adult population of various selected countries.

**Methodology:**

A cross-sectional, quantitative design and analytical study utilized an online survey disseminated from November to December 2021.

**Results:**

A total of 6689 respondents from ten countries participated in the study. The largest number of respondents was from Malaysia (23.9%), followed by Bangladesh (15.5%), Georgia (14.8%), and Turkey (12.2%). The prevalence of social media users was over 90% in Austria, Georgia, Myanmar, Nigeria, and the Philippines. The majority of social media users were from the 18–24 age group. Multiple regression analysis showed that higher education level was positively correlated with all four domains of WHOQoL. In addition, the psychological health domain of quality of life was positively associated in all countries. Predictors among Social Media Needs, Affective Needs (β = -0.07), and Social Integrative Needs (β = 0.09) were significantly associated with psychological health.

**Conclusion:**

The study illuminates the positive correlation between higher education levels and improved life quality among social media users, highlighting an opportunity for policymakers to craft education-focused initiatives that enhance well-being. The findings call for strategic interventions to safeguard the mental health of the global social media populace, particularly those at educational and health disadvantages.

**Table:** 

Text box 1. Contributions to the literature
**• Filling a Gap in Social Media and Quality of Life Research:** This manuscript addresses a void in literature by investigating how perceived social media needs affect adults’ quality of life across ten countries. It broadens understanding of digital communication’s impact on overall well-being.
**• Global Perspective on Social Media Usage and QOL:** Insights into varying social media usage across regions, notably Southeast Asia, Southern Europe, West Asia, and West Africa, enrich our global understanding. This contextualizes digital engagement’s implications for quality of life.
**• Nuanced Age and Gender Dynamics in Usage Patterns:** Contrasting traditional patterns, this research reveals distinctive age and gender-based trends in social media use. Such insights extend discussions on well-being’s intersection with digital platforms.
**• Complex Links between Social Media Needs and Quality of Life Domains:** By connecting diverse social media needs to various quality of life domains, this study uncovers intricate associations. This nuanced understanding adds depth to discussions on digital platforms’ role in shaping well-being.
**• Implications for Public Health Interventions:** This research’s identification of links between social media needs and well-being informs potential interventions. This contribution holds promise for guiding strategies that enhance mental and physical health through tailored approaches to digital platform usage.

## Introduction

The development of internet technology has revolutionized the way people live. As a result, social media has become an integral part of daily life. It is hard to find a person who has internet access but does not use social media. Carr and Hayes [1] defined social media as “Internet-based channels that allow users to interact and selectively self-present opportunistically, either in real-time or asynchronously, with both broad and narrow audiences who derive value from user-generated content and the perception of interaction with others”. Examples of widely used social media platforms include Facebook, LinkedIn, Instagram, WhatsApp, and other apps that enable online social interaction. Over time, the use of social media has increased significantly, primarily for obtaining information, conducting research, creating a social image, interacting with the wider community, and expressing emotions with each other [2].

Furthermore, communities rely heavily on social media as it can change their perception and behaviour according to the information they receive via social media; also, they spend much time using it [3]. On average, users spend worldwide 2.24 hrs per day on social media, 30 min more than in 2015 [4]. In January 2021, 4.2 billion people were using social media globally, which is expected to reach six billion by 2027 [4].

A new paradigm of social interaction has evolved with the arrival of social media. It brought both positive and negative effects on human life. In one aspect, it provided an opportunity to connect with distant and diverse community/family relatives and information sources, allowing close and frequent interaction and an opportunity in helping to solve each other’s emotional and other daily life challenges [5, 6]. Some studies report an increment in quality of life, and some reported no significant improvement [7–9].

The global assimilation of social media into everyday life has ushered in a complex array of impacts on health-related quality of life (HRQoL), exhibiting profound diversity across various cultures and demographics. This variation necessitates a collaborative international policy approach that both recognizes and respects these differences, enabling targeted strategies to mitigate the risks and amplify the benefits of social media on a global scale. It is essential to foster research that highlights cultural nuances to optimize social media’s role in enhancing QoL universally. A study conducted among adolescents in the Netherlands reported decreased HRQoL with the longer use of social media [10]. Particularly, the excessive or inappropriate use of social media is reported to cause more anxiety-like mental health-related problems (stress, anxiety and depression) than minimizing it [11, 12]. The literature has determined that it has affected people’s regular work routine, well-being, happiness and mental health [13, 14]. Furthermore, Oberst et al., 2017, stated that there is a higher potential for using social media among people already suffering from depression and anxiety-like mental illnesses [15]. Additionally, increased mental health-related problems have been linked to higher social media use during the COVID-19 pandemic [16]. The concept of digital well-being was widely discussed during the pandemic, as social media was a major source of information [17].

A recent meta-analysis found insufficient evidence confirming the relationship between well-being and problematic use of social media [12, 18]. During the COVID-19 pandemic, digital health literacy was crucial and linked to improved vaccine confidence and uptake [19–22]. However, beyond digital health literacy, social media usage has certainly impacted the QoL [20]. Rodriguez et al. [23] concluded that the impact of social media differs based on the social media user’s demographic, personality and cultural variances. In addressing this analytical gap, the current research aims to delineate the specific social media needs and their consequential effects on life quality within an international context. Thus, the finding of one location may not accurately reflect the situation of different places of people sufficiently. Despite several studies outlining the negative impact of COVID-19 on health and QoL [24–26], limited evidence is available to examine the impact of social media use on quality of life. There have been only a few global studies documenting the impact of social media on HRQoL. The social media usage has become a pervasive element of human interaction. The handling of social media or the Internet affects the physical, mental, and spiritual health of the people and as such the QoL [7, 9, 27, 28]. Therefore, this study aimed to explore the perceived social media needs and their impact on the QoL among the adult population of various selected countries. Our research introduces novel insights by providing a multi-country analysis that contrasts the effect of social media on QoL in varied cultural contexts, offering a granular understanding of its role across diverse global populations. It is the first of its kind to employ a comparative cross-national approach to examine the interplay between social media needs and life quality post the COVID-19 pandemic, filling a critical gap in existing literature.

## Materials and methods

A quantitative-based cross-sectional study was conducted in the countries Austria, Bangladesh, Georgia, Iran, Iraq, Malaysia, Myanmar, Nigeria, Philippines, Turkey from November 2021 to December 2021. The inclusion criteria for this study were citizens residing in the involved countries, aged 18 years and above, reachable via phone or over the internet, using a network connection, and willing to participate in this study.

The study sample size was calculated using an adjusted single population proportion formula with an additional 30% of the non-response rate, giving rise to the final sample size, *n* = 490. Non-probability convenience sampling will be used for sample collection.

This study used an online questionnaire available in both in their native language and English versions. In addition, three experts did the back-to-back translation. The questionnaire was adapted from validated sources: WHO Quality of Life-BREF [29] and the Social Networking Sites Uses and Needs questionnaire [2]. The online questionnaire consists of 4 sections and a total of 65 items. Section A: Sociodemographic profile (10 items), Section B: Social Networking Sites Usage and Needs (SNSUN) (27 items) and Section C: Quality of Life (WHOQOL-BREF) (26 items). The WHOQOL-BREF questionnaire consists of 26 instruments, of which 24 items are differentiated into four domains, namely physical health (seven items), psychological health (six items), social relationships (three items) and environment (eight items). The WHOQOL-BREF has shown good discriminant validity, content validity, internal consistency, and test–retest reliability [29]. The reliability of Physical health domain, psychological health, social relationship and environment were 0.71–0.79, 0.70–0.74, 0.80–0.87 and 0.81–0.89, respectively. The cut-off point for a predictor of overall good QoL of the WHOQOL-BREF questionnaire is set to be > 60 to maintain sensitivity and positive predictive value [30].

### Statistical analysis

Statistical Package for Social Sciences (SPSS) version 25.0 for windows was used to analyze the data. The continuous variables were expressed as means and standard deviations, while categorical variables were expressed as proportions and frequencies. Bivariate analyses were performed to identify the possible significant factors for the four domains of the WHOQoL scale. An independent sample t-test was performed for two group comparisons. Linear regression was performed to determine the factors associated with the four domains of the WHOQoL scale. A *p*-value of < 0.05 was considered statistically significant in all the analyses.

## Results

A total of 6689 respondents from ten countries participated in the study. The largest number of respondents was from Malaysia (23.9%), followed by Bangladesh (15.5%), Georgia (14.8%), and Turkey (12.2%). The least respondents were from Myanmar (1.2%) and Nigeria (1.8%). Among the subjects, the majority (35.3%) were in the age group between 18 and 24, followed by 25–44 (27.5%). More than half of the respondents were female (51.5%). Around 47% were married, and 45% were single. Maximum (44.7%) respondents were tertiary level education, and most of their income sources were work (46%). Over half were employed (51.5%), and around 40% were not employed. The living arrangements for 81% of respondents were with family, and more than three quarters of the respondents were residing in an urban area. Around 19.4% were living with an illness (Table 1).

**Table 1:** Basic characteristics of the respondents

Variables	Categories	Frequency	Percent
**Country of Residence**	Austria	388	5.8
	Bangladesh	1035	15.5
	Georgia	990	14.8
	Iran	300	4.5
	Iraq	601	9.0
	Malaysia	1596	23.9
	Myanmar	79	1.2
	Nigeria	119	1.8
	Philippines	763	11.4
	Turkey	818	12.2
**Age group**	18–24 years	2364	35.3
	25–44 years	1840	27.5
	45–64 years	1704	25.5
	65 and more	781	11.7
**Gender**	Male	3244	48.5
	Female	3445	51.5
**Marital status**	Single	3003	44.9
	Married	3139	46.9
	Ever married^a^	547	8.2
**Highest qualification**	No education	147	2.2
	Primary	443	6.6
	Secondary	1174	17.6
	Post-secondary	1936	28.9
	Tertiary	2989	44.7
**Income source**	Work	3078	46.0
	Business	632	9.4
	Children	428	6.4
	Parents	1143	17.1
	Others	1020	15.2
	not available	388	5.8
**Employment status**	Employed	3442	51.5
	Not employed	2709	40.5
	Retired	538	8.0
**Living arrangement**	Alone	632	9.4
	Family	5410	80.9
	Others	597	8.9
	Care-centres	50	0.7
**Residential area**	Urban	5113	76.4
	Rural	1188	17.8
	Not available	388	5.8
**Health Condition**	With Illness	1301	19.4
	Without Illness	5000	74.7
	Not available	388	5.8
**Total**		**6689**	**100**

^a^_Ever married -Divorced/Widow/Single Parent_

**Fig. 1:**
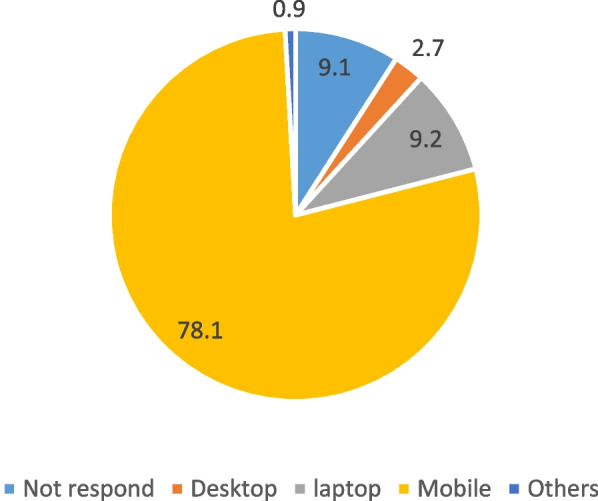
Device prefer to use social media

Table 2 demonstrates the prevalence of social media users. Age group, gender, marital status, highest qualification, Income source, employment status, living arrangements, residential area, and health condition were statistically significant with social media use. The prevalence of social media users was over 90% in Austria, Georgia, Myanmar, Nigeria, and the Philippines. The age-wise majority of uses of social media was higher in the age group 18–24. However, social media services among males were higher (91.8%) than for females (90%). Marital status as a ‘single’ was more prevalent (97%), and tertiary education (95.6%) reported a higher social media use. In addition, the prevalence was higher (97.3%) among respondents who are financially dependent on the parents. Also, employed respondents had a higher prevalence (94.5%) of social media use compared to unemployed (88.00). Respondents with living arrangements with family also reported higher use of social media. Likewise, those staying in the urban area, and those without illnesses (93%), had a higher prevalence of social media use. About 388 respondents’ did not report their income source, residential area, and health condition. Figure 1 represents the device that prefers to use social media. Most respondents used mobile devices (78.1%), followed by laptops or notebooks (9.2%) for social media use.

**Table 2:** Prevalence of social media users

	**No**	**Yes**	**n**	**χ**^**2**^**, *p* value**
**Country**				
Austria	0.0	100.0	388	
Bangladesh	13.3	86.7	897	
Georgia	3.4	96.6	956	
Iran	16.0	84.0	252	
Iraq	13.0	87.0	523	
Malaysia	12.7	87.3	1393	
Myanmar	3.8	96.2	76	
Nigeria	0.0	100.0	119	
Philippines	1.0	99.0	755	
Turkey	11.6	88.4	723	
**Age group**				
18–24 years	3.70	96.30	2364	602.758, < 0.001
25–44 years	5.20	94.80	1840	
45–64 years	10.40	89.60	1704	
65 and more	31.60	68.40	781	
**Gender**				
Male	8.20	91.80	3244	5.843,0.016
Female	9.90	90.10	3445	
**Marital status**				
Single	3.00	97.00	3003	653.005, < 0.001
Married	10.60	89.40	3139	
Divorced	15.00	85.00	180	
Widow	44.10	55.90	295	
Single parents	36.10	63.90	72	
**Highest qualification level**				
No education	47.60	52.40	147	754.545, < 0.001
Primary	35.20	64.80	443	
Secondary	12.20	87.80	1174	
Post-secondary	5.50	94.50	1936	
Tertiary	4.40	95.60	2989	
**Income source**				
Work	7.40	92.60	3078	520.032, < 0.001
Business	7.80	92.20	632	
Children	36.20	63.80	428	
Parents	2.70	97.30	1143	
Others	14.10	85.90	1020	
Not available	0.0^a^	100.00	388	
**Employment status**				
Employed	5.50	94.50	3442	125.58, < 0.001
Not employed	12.00	88.00	2709	
Retired	17.30	82.70	538	
**Living arrangement**				
Alone	9.70	90.30	632	50.21, < 0.001
Family	8.50	91.50	5410	
Others	11.40	88.60	597	
Carecenters	36.00	64.00	50	
**Residential area**				
Urban	9.20	90.80	5113	48.68, < 0.001
Rural	11.70	88.30	1188	
Not available		100.00	388	
**Health Condition**				
With Illness	20.20	79.80	1301	263.60, < 0.0001
Without Illness	6.90	93.10	5000	
Not available		100.00	388	
**Total**	9.10	90.90	6689	

^a^_0 cell, Exact p value cannot be computed_

Table 3 shows the frequency of selected social networking sites. Over half (52%) of the participants used Facebook daily, while only 8.1% used Twitter. WhatsApp was used by 44.8% every day. More than one-third (37.4%) of the respondents used Instagram daily, 44.8 % used YouTube daily, and 35.3% of participants Google every day.

**Table 3:** Uses of social networking sites by users

	**Not Respond**	**Never**	**Rarely**	**Occasionally**	**3-5timesaday**	**Everyday**
**Facebook**	607	677	281	648	1003	3473
	9.1	10.1	4.2	9.7	15.0	51.9
**Twitter**	607	3257	772	909	603	541
	9.1	48.7	11.5	13.6	9.0	8.1
**WhatsApp**	607	883	404	747	1050	2998
	9.1	13.2	6.0	11.2	15.7	44.8
**Instagram**	607	1195	400	857	1129	2501
	9.1	17.9	6.0	12.8	16.9	37.4
**YouTube**	607	528	204	772	1579	2999
	9.1	7.9	3.0	11.5	23.6	44.8
**Google**	995	1258	363	653	1061	2359
	14.9	18.8	5.4	9.8	15.9	35.3
**Others**	995	1401	581	1052	1112	1548
	14.9	20.9	8.7	15.7	16.6	23.1

Table 4 presents perceived social media needs and QoL among participants. The mean score of social media needs were 8.0 (3.15), 11.31 (4.32), 7.1 (3.09), 9.4 (3.98), and 14.39 (5.22) for the diversions, cognitive needs, affective needs, personal integrative and integrative social needs respectively. Almost 39.8 and 43.2 percent of the participants self-reported poor QoL and poor health satisfaction (a score less than four is considered a poor QoL and poor health satisfaction). The mean score of the perceived QoL for domains was 61.38 (15.73), 59.36 (16.98), 57.93 (24.15), and 60.3 (18.72) for the physical health, psychological health, social relationship, and environments domain, respectively.

**Table 4:** Perceived social media needs and quality of life among participants

	Mean ± SD or %	N
**Social Media Needs**		
Diversions: (Escapism and Tension Release)	8.0 ± 3.15	6689
Cognitive Needs (Acquire Information & Knowledge)	11.31 ± 4.32	6689
Affective Needs (Emotions, Pleasure & Feelings)	7.10 ± 3.09	6689
Personal Integrative (Enhance credibility, status)	9.41 ± 3.98	6689
Social Integrative Needs (Interaction with Friends and Family)	14.39 ± 5.22	6689
**Quality of life**		
Quality of life		
Score 1	5.3	356
2	10.9	728
3	23.6	1578
4	48.6	3250
5	11.6	777
Health satisfaction		
Score 1	4.8	320
2	13.5	902
3	24.9	1666
4	44.6	2982
5	12.2	819
Physical health domain	61.38 ± 15.73	6689
Psychological health domain	59.36 ± 16.98	6689
Social relationship domain	57.93 ± 24.15	6689
Environment domain	60.3 ± 18.72	6689

Table 5 presents the relationship between social media needs and QoL by country. The average physical QoL was the highest in Nigeria (68.55 ± 13.24) and lowest in Austria (55.31 ± 10.24). Similarly, psychological QoL was also higher in Nigeria (69.24 ± 13.46) and lowest in Austria (51.04 ± 10.15). Social relationship QoL was higher in Austria (73.47 ± 18.49) and lowest in Iran (53.65 ± 23.99). Furthermore, the environment QoL was highest in Nigeria (69.2 ± 15.87) and the lowest in Iran (54.2 ± 20.13).

**Table 5:** Relation between social media needs and quality of life

Variables	*Physical Health* Mean ± SD	*Psychological health* Mean ± SD	*Social relationship* Mean ± SD	*Environment* Mean ± SD	N
*Country*					
Austria	55.31 ± 10.24	51.04 ± 10.15	73.47 ± 18.49	64.6 ± 11.01	388
Bangladesh	62.93 ± 15.26	61.68 ± 16.00	56.72 ± 24.45	62.1 ± 17.38	1035
Georgia	60.89 ± 16.35	59.04 ± 17.92	56.82 ± 23.93	58.8 ± 19.62	990
Iran	58.12 ± 15.92	54.52 ± 17.82	53.65 ± 23.99	54.2 ± 20.13	300
Iraq	59.34 ± 16.58	57.58 ± 17.82	56.21 ± 24.66	56.7 ± 19.93	601
Malaysia	63.73 ± 14.76	62.5 ± 15.85	59.69 ± 22.71	63.4 ± 17.41	1596
Myanmar	60.78 ± 16.53	57.14 ± 15.10	54.34 ± 22.86	58.2 ± 18.38	79
Nigeria	68.55 ± 13.24	69.24 ± 13.46	64.50 ± 23.27	69.2 ± 15.87	119
Philippines	61.81 ± 16.57	57.71 ± 18.20	54.05 ± 25.01	58.1 ± 20.10	763
Turkey	59.57 ± 16.74	58.07 ± 17.68	55.83 ± 25.02	57.8 ± 20.16	818
*Diversion*					
Negative	59.53 ± 15.20	57.67 ± 15.90	60.97 ± 23.88	60.45 ± 18.61	725
Positive	61.87 ± 15.63	59.88 ± 16.96	57.83 ± 24.05	60.48 ± 18.72	5357
*P* value	< 0.001	0.001	0.001	0.961	
*Cognitive Needs*					
Low	57.79 ± 14.98	55.20 ± 16.72	58.75 ± 24.20	57.82 ± 18.34	507
High	61.94 ± 15.61	60.02 ± 16.81	58.15 ± 24.04	60.72 ± 18.72	5575
*P* value	< 0.001	< 0.001	0.595	0.001	
*Affective Needs*					
Low	61.67 ± 14.94	59.79 ± 16.00	61.24 ± 23.34	61.84 ± 17.85	1813
High	61.56 ± 15.87	59.54 ± 17.20	56.91 ± 24.24	59.9 ± 19.03	4269
*P* value	0.799	0.600	< 0.001	< 0.001	
*Personal Integrative*					
Low	61.73 ± 15.21	59.89 ± 16.57	59.9 ± 23.65	61.89 ± 18.02	1596
High	61.55 ± 15.73	59.52 ± 16.95	57.6 ± 24.17	59.98 ± 18.92	4486
*P* value	0.685	0.445	0.001	< 0.001	
*Social Integrative Needs*					
Low	56.8 ± 14.81	54.56 ± 16.10	62.31 ± 23.45	59.68 ± 15.60	176
High	61.74 ± 15.60	59.77 ± 16.85	58.08 ± 24.06	60.5 ± 18.79	5906
*P* value	< 0.001	< 0.001	0.022	0.564	

Those who used social media for diversion were statistically significant in all three QoL domains. In addition, they were significantly associated with physical, psychological, and social QoL. Those who used social media for cognitive needs were significantly associated with the physical, psychological, and environmental domains of QoL compared with those who did not use it. Those who used social media for affective needs were statistically significant for social and environmental QoL. Social media used for personal integrative or to enhance credibility and status were statistically significant in the social relationship domain of QoL (*p* = 0.001) and environmental QoL (*p* < 0.001). However, social media needs for social integrative needs or interaction with friends and family were statistically significant in three domains physical (*p* < 0.001), psychological (< 0.001), and social relationship (*p* = 0.022).

In the multiple regression analysis (Table 6), all the determinants were included together, where the dependent variable was all four domains of QoL. All countries were positively associated with the physical and psychological health domain. Similarly, all countries except Malaysia and Nigeria were not significantly associated with the environmental QoL.

**Table 6:** Multiple regression analysis is performed with 4 domains of WHO QOL index value (Continuous) as dependent variable and socio -demographic variable with social media needs as the independent variable

**Predictors**	**Physical health**		**Psychological health**		**Social relationship**		**Environment**	
	**Std. β (95%CI)**	***P* value**	**Std. β (95%CI)**	***P* value**	**Std. β (95%CI)**	***P* value**	**Std. β (95%CI)**	***P* value**
**Austria**	**Ref**		**Ref**		**Ref**		**Ref**	
Bangladesh	0.189 (6.01–10.621)	< 0.001	0.22 (8.026; 12.913)	< 0.001	-0.259 (-21.099; -14.037)	< 0.001	-0.063 (-6.047; -0.569)	0.018
Georgia	0.151 (4.217–8.76)	< 0.001	0.169 (5.406; 10.221)	< 0.001	-0.264 (-20.897; -13.94)	< 0.001	-0.12 (-8.855; -3.458)	< 0.001
Iran	0.044 (0.712–6.136)	0.013	0.051 (1.465; 7.214)	0.003	-0.165 (-24.04; -15.734)	< 0.001	-0.109 (-13.477; 7.032)	< 0.001
Iraq	0.071 (1.565–6.352)	0.001	0.113 (4.277; 9.35)	< 0.001	-0.216 (-22.186; -14.855)	< 0.001	-0.132 (-11.68; -5.993)	< 0.001
Malaysia	0.235 (6.478–10.996)	< 0.001	0.314 (10.213; 15.002)	< 0.001	-0.255 (-18.038; -11.118)	< 0.001	-0.029 (-3.956; 1.412)	0.353
Myanmar	0.036 (1.147–9.064)	0.011	0.036 (1.233; 9.625)	0.011	-0.1 (-27.664; -15.539)	< 0.001	-0.048 (-12.715; -3.309)	0.001
Nigeria	0.11 (9.008–15.845)	< 0.001	0.136 (12.951; 20.198)	< 0.001	-0.067 (-16.839; -6.368)	< 0.001	0.016 (-1.887; 6.236)	0.294
Philippines	0.132 (3.816–8.652)	< 0.001	0.103 (2.722; 7.848)	< 0.001	-0.302 (-25.708; -18.302)	< 0.001	-0.15 (-11.364; -5.618)	< 0.001
Turkey	0.081 (1.581–6.233)	0.001	0.13 (4.294; 9.225)	< 0.001	-0.262 (-23.061; -15.938)	< 0.001	-0.145 (-11.137; -5.61)	< 0.001
Age	0.03 (-0.019; 0.076)	0.238	0.012 (-0.038; 0.063)	0.632	0.073 (0.034; 0.18)	0.004	0.053 (0.004; 0.117)	0.036
**Female**	**Ref**		**Ref**		**Ref**		**Ref**	
Male	0.012 (-0.444; 1.177)	0.376	0.032 (0.206; 1.924)	0.015	0.018 (-0.364; 2.118)	0.166	0.037 (0.409; 2.335)	0.005
**Ever married**	**Ref**		**Ref**		**Ref**		**Ref**	
Single	-0.035 (-3.122; 0.922)	0.286	0.027 (-1.246; 3.04)	0.412	0.048 (-0.786; 5.407)	0.144	0.002 (-2.331; 2.474)	0.954
Married	-0.023 (-2.469; 1.053)	0.431	0.025 (-1.035; 2.698)	0.382	0.02 (-1.733; 3.66)	0.484	-0.021 (-2.898; 1.286)	0.45
**No formal education**	**Ref**		**Ref**		**Ref**		**Ref**	
Primary	0.023 (-2.193; 5.644)	0.388	0.073 (1.65; 9.957)	0.006	0.045 (-0.866; 11.136)	0.094	0.062 (0.776; 10.087)	0.022
Secondary	0.088 (0.048; 7.237)	0.047	0.16 (3.358; 10.979)	< 0.001	0.108 (1.425; 12.436)	0.014	0.15 (3.187; 11.729)	0.001
Postsecondary	0.093 (-0.402; 6.695)	0.082	0.158 (2.04; 9.563)	0.003	0.112 (0.448; 11.316)	0.034	0.163 (2.421; 10.852)	0.002
Tertiary	0.125 (0.371; 7.44)	0.03	0.199 (2.974; 10.467)	< 0.001	0.149 (1.75; 12.577)	0.01	0.207 (3.575; 11.974)	< 0.001
**Work**	**Ref**		**Ref**		**Ref**		**Ref**	
Business	0.029 (0.118; 3.006)	0.034	0.039 (0.707; 3.769)	0.004	0.019 (-0.656; 3.768)	0.168	0.029 (0.152; 3.583)	0.033
Children	-0.001 (-2.346; 2.194)	0.947	0.022 (-0.595; 4.217)	0.14	0.007 (-2.686; 4.266)	0.656	0.014 (-1.433; 3.961)	0.358
Parents	0.029 (-0.345; 2.671)	0.131	0.07 (1.459; 4.655)	< 0.001	0.045 (0.481; 5.1)	0.018	0.053 (0.756; 4.339)	0.005
Others	0.017 (-0.62; 2.122)	0.283	0.034 (0.174; 3.082)	0.028	0.029 (-0.099; 4.102)	0.062	0.02 (-0.577; 2.682)	0.205
**Employed**	**Ref**		**Ref**		**Ref**		**Ref**	
Not employed	-0.003 (-1.297; 1.089)	0.864	-0.043 (-2.748; -0.219)	0.021	0.021 (-0.797; 2.856)	0.269	-0.002 (-1.51; 1.325)	0.898
Retired	-0.013 (-2.536; 0.987)	0.389	-0.034 (-4.076; -0.343)	0.02	-0.019 (-4.471; 0.923)	0.197	-0.033 (-4.453; -0.268)	0.027
**Alone**	**Ref**		**Ref**		**Ref**		**Ref**	
Family	0.041 (0.268; 3.024)	0.019	0.011 (-0.998; 1.923)	0.534	0.015 (-1.16; 3.06)	0.378	0.011 (-1.094; 2.18)	0.515
Others	0.047 (0.736; 4.474)	0.006	0.052 (1.14; 5.101)	0.002	0.042 (0.722; 6.445)	0.014	0.051 (1.174; 5.614)	0.003
CareCentres	-0.041 (-14.328; -3.377)	0.002	-0.053 (-18.161; -6.554)	< 0.001	-0.02 (-15.049; 1.721)	0.119	-0.055 (-20.735; -7.724)	< 0.001
**Rural**	**Ref**		**Ref**		**Ref**		**Ref**	
Urban	-0.002 (-1.139; 1.009)	0.905	0.002 (-1.056; 1.221)	0.887	0.026 (-0.198; 3.092)	0.085	0.016 (-0.562; 1.991)	0.272
**Without Illness**	**Ref**		**Ref**		**Ref**		**Ref**	
With Illness	-0.09 (-4.851; -2.569)	< 0.001	-0.141 (-7.506; -5.088)	< 0.001	-0.113 (-8.967; -5.473)	< 0.001	-0.128 (-7.738; -5.027)	< 0.001
**Social Media Needs**								
Diversion	0.009 (-0.174; 0.314)	0.575	0.015 (-0.128; 0.389)	0.322	0.001 (-0.358; 0.389)	0.937	0.016 (-0.14; 0.44)	0.31
Cognitive Needs	0.01 (-0.139; 0.262)	0.547	0.01 (-0.143; 0.281)	0.525	0.024 (-0.076; 0.538)	0.14	0.02 (-0.09; 0.386)	0.224
Affective Needs	-0.073 (-0.759; -0.265)	< 0.001	-0.076 (-0.837; -0.314)	< 0.001	-0.076 (-1.198; -0.443)	< 0.001	-0.07 (-0.883; -0.296)	< 0.001
Personal Integrative	0.013 (-0.118; 0.26)	0.46	0.004 (-0.176; 0.224)	0.816	0.007 (-0.227; 0.351)	0.674	-0.001 (-0.231; 0.217)	0.952
Social Integrative Needs	0.07 (0.231; 0.585)	< 0.001	0.09 (0.375; 0.75)	< 0.001	0.065 (0.308; 0.85)	< 0.001	0.082 (0.361; 0.781)	< 0.001

Secondary (β = 0.08) and tertiary educated respondents (β = 0.125), whose work was ‘business’ (β = 0.02) were not significantly associated with the physical health psychological health, social relationship, and environmental QoL. Living with family (β = 0.04), and ‘other’ living arrangements (β = 0.047) were positively associated with physical health domain of QoL. However, living in care centres (β = -0.041) and having an illness (β = -0.09) were negatively related to physical health quality. Social media needs for affective needs (β = -0.073) and social integrative needs (β = 0.07) were significantly associated with the physical health domain of QoL.

The psychological health domain of QoL was positively associated in all countries. Sociodemographic predictors for psychological health domain of QoL showed that male gender (β = 0.03), primary (β = 0.07), secondary (β = 0.16), postsecondary (β = 0.16), and tertiary level of education (β = 0.19) were positively associated. In addition, those working in ‘business’ (β = 0.03), and whose parents were working (β = 0.07) and doing ‘other’ work (β = 0.03), living with ‘others’ (β = 0.05) were positively associated with the psychological health domain of QoL.

Those who are not employed (β = -0.04) and retired (β = -0.03), or reported to live in care centres (β = -0.05), had an illness (β = -0.14), showed a negative association with the psychological health domain of QoL.

Predictors among social media needs, Affective Needs (β = -0.07), and Social Integrative Needs (β = 0.09) were significantly associated with psychological health. All participating countries were negatively associated with social relationships. The social relationship is positively associated with age (β = -0.07), secondary education level (β = -0.10), Postsecondary (β = 0.11) and tertiary level education (β = 0.149), and parents were working (β = 0.04), Living with ‘Others’ (β = 0.04), and having an illness (β = -0.11). Affective needs for social media (β = -0.07) were negatively associated with the social relationship domain of QoL. However, Social Integrative Needs (β = 0.065) were positively related to social relationships. Age (β = 0.05), male gender (β = 0.03), primary (β = 0.06), secondary (β = 0.15), postsecondary (β = 0.16), and tertiary (β = 0.207) level of education, working in business (β = 0.03), Working parents(β = 0.05), retirement status(β = -0.03), Living with ‘Others’(β = 0.05), living in care centres (β = -0.05), having illness (β = -0.128) were positively associated with environment QoL. However, among social media needs, affective Needs (β = -0.07) were negatively related to the environmental health domain of QoL, and integrative social needs (β = 0.08) were positively associated with environment.

## Discussion

Our world today is undeniably digital. Social media has become the go-to guide for over 61.4 percent of the global population. Despite the widespread use of social media among people of all ages, limited studies have explored the impact of social media on the overall quality of life (QoL) of populations [7, 9, 27, 28]. Specifically, this study sought to fill this gap by assessing the perceived social media needs and QoL among the adult population across ten countries.

For country statistics, our study findings showed that the percentage of social media users was highest in regions of Southeast Asia (Myanmar, Philippines), Southern Europe (Austria), West Asia (Georgia) and West Africa (Nigeria), whereas the lowest number of social media users was reported in the Middle East (Iran, Iraq). These results were aligned with the Global Social Media Research Summary 2021/2022, which ranked Southeast Asia - sixth, Southern Europe - seventh, and West Asia – ninth for the highest social network penetration rate [31].

In terms of sociodemographic, among 6689 participants recruited in this study, over one-third were young adults ranged from 18–24 years. According to previous studies conducted in United States in 2015, the mean age of respondents was 28.8 years old, suggesting the usage of social media among working age group [32]. As compared to our study conducted in 2021, is seen increasing trend for young adults’ social media users. Similarly, the in Global Social Media Research Summary 2021/2022 found that Generation Z aged 10–25 showed an increasing trend in social media use [31]. Generation X and Millennials aged 26–57 showed a decreasing trend in social media usage due to increasing real-life responsibilities and an increasing trend for the Boomer generation as social media allows connection and communication with the younger generation [31]. A systematic review conducted on social media sites and older users also shows the ability for intergenerational communication is the main driving factor for the elderly to use social media sites [33]. This study also found that social media usage was slightly higher in males than females. Consistent with the Global Social Media Research Summary 2021/2022, male users predominate social media usage across all age ranges except those aged 45 years and above [31].

Interestingly, our study findings suggested that the most used social media platforms were Facebook and its associate media sites, WhatsApp, and Instagram, which are under the parent company - Meta. These findings were consistent with the Global Social Media Research Summary 2021/2022, indicating that Facebook was the most visited social media platform, predominantly visited by those aged 58 years and above [31]. Google was ranked the first most visited website worldwide, and its subsidiary company YouTube remains the top video-sharing site. YouTube and Instagram are mostly visited by those ages 18–24 at 89% and 74%, respectively. Contrary to the Global Social Media Research Summary 2021/2022, Twitter was the second most used social media platform compared to our study that showed Twitter had the least usage [31].

Country-wise QOL assessment, this study found that the mean scores for perceived QOL were lower in all domains compared to Portugal [34]; lower in psychological health and social relationship domains compared to Brazil [35] and higher for physical and environmental health domains than Brazil and Malawi [35, 36]. Despite our study deduced that Nigerians perceived higher QOL than Malaysian and Turkish people in all domains, Skevington et al. found contradictory findings [37]. Except the social health domain was in line with our study, the mean score for the physical health domain was higher in Malaysia than in Nigeria and Turkey. Similarly, the mean score for the psychological health domain in Malaysia and Nigeria were equally higher than in Turkey. Furthermore, they also revealed that environmental health domain scores were higher in Malaysia than in Turkey and Nigeria [37]. However, it is noteworthy that these comparisons are interpreted with due caution as a previous study showed that physical and psychological domains of WHOQOL-BREF were less invariant than social relationship and environmental domains. Only 11 out of 24 facet items, excluding four facets that were fixed as reference items for which their invariance could not be assessed, were found to have invariant factor loadings and thresholds in the study mentioned above [38]. Alarming as it may sound, meaningful comparisons still can be made, provided that the proportion of non-invariant items is rather small [38].

Multiple regression analysis of sociodemographic backgrounds and four domains of WHOQOL index value showed that higher education level was positively correlated with all four domains of WHOQOL-BREF. Likewise, previous studies also reported that education level was significantly associated with physical, psychological, social relationship and environment health domain [34, 35, 39]. In our study, living with family and others led to better physical health scores than living alone. These findings were consistent with a previous study conducted by Patrício et al. in 2014, suggesting that living with parents, partners, or children could result in better physical health [34]. Contrary, existing literature proved unequivocally that living alone was linked deleteriously to a rise in blood pressure, poorer sleep quality, detrimental effects on immune stress response and deterioration in cognition levels over time in the elderly, which can ultimately jeopardize overall physical health [40].

In line with previous studies, gender was also determined as one of the predictors for the psychological health domain in our study, in which males were found to have better QoL than females [31, 35, 41]. However, controversial results were also found in some studies, ascertained that gender was not correlated with psychological health [39, 42]. Our findings could be attributed to women’s multiple social burdens of being wives, mothers or carers, single parents or widows and the effects of their vulnerability to domestic and sexual violence [43]. Another study on older women living in low, densely populated areas in the central southern region of Portugal also shows that they are susceptible to ageing and exhibit a greater dependency on their loved ones, making them vulnerable to psychological and physical health [44].

Our study also revealed that employment status is related to psychological health, in which employed individuals had better psychological health than those who were unemployed. Similar findings were found in two studies which suggested that employment influences the QoL of the general population [31, 34]. However, existing literature also argues that retired individuals have better psychological health than employed individuals, mainly due to workplace violence experience, poor psychosocial job quality and low job control [45, 46]. Meanwhile, a possible explanation for our finding is that unemployment leads to the deprivation of several latent functions of employment, such as financial strain, social contacts, time structure and personal status or identity in institutions, which are also fundamental psychological needs that are important for mental health [47]. Moreover, prolonged uncertainty, self-doubt and anxiety among those unemployed also lead to a further decrement in psychological health.

In addition, our study also found that living with illness and in care centres were negatively correlated with psychological health. This finding is in accordance with a previous study conducted by Ghasemi et al. [48], suggested that older adults who prefer to live with their families could have better QoL. However, in contrast, Chung found that community-dwelling elderly had 3.14 higher odds of depression compared to nursing home elderly [49]. Nevertheless, poor psychological health among those living in residential homes could be due to the loss of freedom, social status, autonomy and self-esteem, neglect from children and approaching death [50]. As for people with illnesses, similar to our findings, numerous literatures have suggested that living with illness can affect moods, emotions, behaviour of a person, and eventually leading to poorer mental health [31, 34, 35, 39].

Other than that, there was a positive association between age and environment QoL. Previous study supported the idea that personal and national ageing encourages individual pro-environmental behaviour [51], which is consistent with the theory of generativity. As people age, they may increasingly seek self-transcendence and meaning in life and pursue pro-social goals, and the practice of environmentally friendly actions may become one way for older persons to impart such wisdom. Besides, older people may become more involved in environmental issues due to their enhanced perceived effects of environmental risks on human health [51]. Furthermore, our findings on the positive association between education and environmental health was supported by another study, which suggested that decreasing the number of secondary school dropouts might increase pro-environmental behaviour [52]. The possible reason was that additional education explicitly teaches people the value of the environment [52].

As for social media needs, our study revealed that affective and social integrative needs were significantly associated with the physical health domain of QoL. According to previous research, people who actively engage in online social networks were more likely to be socially active by having online interactions and new friends. This may have favourable effects on their physical well-being [53]. Controversially, previous literature also found strong feelings of dependency on Facebook was correlated with poorer physical health [54].

Moreover, our study revealed that affective and social integrative needs were significantly associated with psychological health. In fact, it is known that humans genetically have a strong desire to connect with people, especially to share their feelings. By utilizing social media, users who enjoy virtual connections would gain many advantages, which could potentially affect their emotional well-being and psychological health [55]. In line with our study, previous research revealed a positive correlation between online social media use for interaction and psychological health [56]. Indeed, social media can provide opportunities to engage and support individuals with mental health issues [57]. Contrary, a systematic review of 16 studies found a negative association between social integrative needs and psychological health. It found that some teens had anxiety from social media due to fear of missing out, and they would regularly check all their friends’ messages [58]. In addition, a recent study revealed that taking a 1-week break from using social media can substantially improve well-being, depression, and anxiety [59].

Interestingly, for social relationship domain of QoL, our study findings suggested that it has a negative correlation with affective needs, whereas a positive association with social integrative needs. This might be due to social media use for affective needs often produces unrealistic expectations as people may compare their physical and virtual relationships [60]. Another possible reason was that certain characteristics of social media users like social isolation might influence real-life social relationship quality. However, particularly for students with introvert personality, they were more likely to communicate online as online chatting is more comfortable for them [61]. In addition, our finding could be attributable to the benefit of relational reconnection from social media, in which social media use can improve social connectedness especially during COVID-19 lockdowns [62, 63]. Preventive measures and practices towards COVID-19 have restrained physical contacts and meetings, which highlighted the crucial need for social media platform in communication [64]. In fact, social media has been the platform for promoting health and disseminating health information globally during the pandemic [20, 28]. Infectious diseases will continue to emerge and re-emerge, leading to unpredictable epidemics and difficult challenges to public health [65, 66]. As going digital is indispensable, this underscores the importance of social media in daily needs fulfillment to enable better well-being and QoL.

Furthermore, the impact of social media use on physical, psychological, and social QoL was found to be statistically significant when used for diversion, aligning with earlier findings that problematic use of social networking sites correlates with attempts to alleviate boredom [67]. Studies have also linked problematic use of social media with poor psychological health outcomes [68], depression [69], and anxiety [70]. The biopsychosocial paradigm—encompassing withdrawal, conflict, tolerance, salience, mood modification, and relapse—provides a framework for understanding problematic social media use [71]. Social media, when used to alter mood or escape problems, can lead to addictive behaviors. The obsession with social media, reflected in its salience, may contribute to sedentary habits and lower levels of physical activity, which increase the risk of non-communicable diseases [72]. Additionally, excessive use can lead to irritability in the absence of social media, potentially harming social interactions [60]. It is imperative for government policies to target the resultant sedentary lifestyles and mental health issues arising from social media use. Moreover, the promulgation of such policies via social media channels is advisable to ensure broad dissemination and enhance the efficacy of government-public communication [73].

### Strengths and limitations

The study leverages a large and culturally diverse sample from 10 countries, enhancing the understanding of social media’s effects on QoL on an international scale. The use of well-validated instruments, the WHOQOL-BREF and SNSUN scales, adds rigor to the research outcomes. It also thoughtfully considers the influence of education on QoL, providing nuanced insights into the social implications of social media use. The study is, however, limited by a convenience sampling method that may not be representative of the global population, potentially biasing the results. Unequal sample sizes across countries pose a challenge for valid cross-cultural comparisons and understanding the differential impact of social media. The cross-sectional design limits the ability to track changes over time or establish causality. Recommendations for future research include adopting probability sampling methods to improve representativeness and balance. Ensuring equal sample distribution across participating countries will enhance the validity of international comparisons. Longitudinal studies are suggested to better understand the causal relationships between social media use and QoL over extended periods.

## Conclusions

Social media usage has become a pervasive part of individuals interaction. Intensive handling and interaction affect the physical, mental, and spiritual health of the people and as such the QoL. This study aimed to explore the perceived social media needs and their impact on the QoL among the adult population of various selected countries. A significant proportion of the survey population reported poor QoL and poor health satisfaction. Physical and psychological QoL was poor among Austrian people, whereas social relationship QoL was higher in Austria. Furthermore, social relationship QoL and environmental QoL was lower among the Iranian population, and this can be tackled by disseminating appropriate policy interventions. Those with illness reported poor physical health quality and it is important to adopt a holistic approach to tackle the problems of those already battling with illness. Finally, higher education acts as a safety net against psychological health; therefore, uneducated or low educated need intrinsic focus to tackle the menace of psychological health. As to what they can do to resolve the issue of low physical and psychological QoL. The significance of these findings lies in their ability to support additional study on social media, mental health and physical and psychological QoL. This finding may interest policymakers to address this topic to public health, in higher boards, companies, and educational sectors.

## Data Availability

The data presented in this study are available on request from the corresponding author.
